# Tasting the *terroir* of wine yeast innovation

**DOI:** 10.1093/femsyr/foz084

**Published:** 2019-12-12

**Authors:** I S Pretorius

**Affiliations:** ARC Centre of Excellence in Synthetic Biology, Macquarie University, 19 Eastern Road, North Ryde, Sydney, NSW 2109, Australia

**Keywords:** avatar yeast, biogeography, microbiome, non-*Saccharomyces*, *Saccharomyces cerevisiae*, synthetic genomics, microbial *terroir*, wine yeast, yeastome

## Abstract

Wine is an archetypal traditional fermented beverage with strong territorial and socio-cultural connotations. Its 7000 year history is patterned by a *tradition* of *innovation*. Every value-adding innovation − whether in the vineyard, winery, supply chain or marketplace − that led to the *invention* of a new *tradition* spurred progress and created a brighter future from past developments. In a way, wine *traditions* can be defined as *remembered innovations* from the distant past − inherited knowledge and wisdom that withstood the test of time. Therefore, it should not be assumed *a priori* that tradition and innovation are polar opposites. The relations between the forces driven by the *anchors of tradition* and the *wings of innovation* do not necessarily involve displacement, conflict or exclusiveness. Innovation can strengthen wine tradition, and the reinvention of a tradition-bound practice, approach or concept can foster innovation. In cases where a paradigm-shifting innovation disrupts a tradition, the process of such an innovation transitioning into a radically new tradition can become protracted while proponents of divergent opinions duke it out. Sometimes these conflicting opinions are based on fact, and sometimes not. The imperfections of such a debate between the ‘ancients’ and the ‘moderns’ can, from time to time, obscure the line between myth and reality. Therefore, finding the right balance between *traditions worth keeping* and *innovations worth implementing* can be complex. The intent here is to harness the creative tension between *science fiction* and *science fact* when innovation's first-principles challenge the *status quo* by re-examining the foundational principles about a core traditional concept, such as *terroir*. Poignant questions are raised about the importance of the *terroir* (biogeography) of yeasts and the value of the microbiome of grapes to wine quality. This article imagines a metaphorical *terroir* free from cognitive biases where diverse perspectives can converge to uncork the effervescent power of territorial yeast populations as well as ‘nomadic’ yeast starter cultures. At the same time, this paper also engages in *mental time-travel*. A future scenario is imagined, explored, tested and debated where *terroir*-less *yeast avatars* are equipped with designer genomes to safely and consistently produce, individually or in combination with region-specific wild yeasts and or other starter cultures, high-quality wine according to the preferences of consumers in a range of markets. The purpose of this review is to look beyond the horizon and to synthesize a link between *what we know now* and *what could be*. This article informs readers where to *look* without suggesting what they must *see* as a way forward. In the context of one of the world's oldest fermentation industries − steeped in a rich history of tradition and innovation − the mantra here is: *respect the past*, *lead the present* and *secure the future* of wine.

## Teaser

The future of wine yeast innovation is likely to uncork the combined effervescent power of territorial wine grape microbiomes, nomadic starter cultures and yeast avatars with no real terroir

## THE PAST AND FUTURE OF WINE IS EVER PRESENT IN ITS TRADITION OF INNOVATION

**Figure 1.**
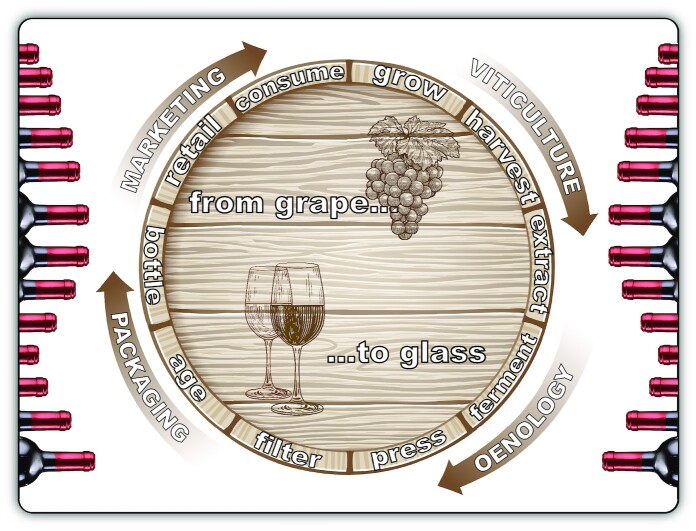
The *from-grapes-to-glass value chain* in wine production. The traditional *production-driven* view of the *supply chain* has been largely replaced by a more innovative *market-driven* approach. This new mindset among contemporary vintners has placed their products in the high-tension field between the forces of *market-pull* and *technology-push*, where tradition and innovation must co-exist.

Product innovation is a vital source of competitive advantage and wine is no exception to this fundamental business principle. The history of wine is marked by a *tradition of innovation* driven by a culture of *innovation through tradition*. For seven millennia, vintners have explored new regions, lands and continents to selectively grow grape cultivars with different properties. New ideas, practices and technological interventions have been applied to optimise these properties for maximum wine quality outcomes. With every new vintage, grapegrowing and winemaking practices were tweaked, tested and learned from. Every successful, value-adding innovation in the vineyard or winery that improved wine quality invented a new tradition. Thus, one could think of innovation as the ancestor of tradition. In each instance when *hindsight* turned into *insight*, and insight into *foresight*, yesteryear's innovation morphed into this year's tradition − a cyclical story of aspiration and ingenuity written by time. Steering towards the future, it is therefore likely that vintners will continue to be inspired by a custom to innovate through tradition. In this context, the value proposition of any future innovation at any point across the entire *from-grapes-to-glass* value chain (Fig. 1) will be critically assessed by producers and consumers through a lens tinted by a deep *respect for the past*, an indomitable spirit to *lead the present* and a quest to *secure the future* of wine. This article recognises that these three core drivers of progress in the ancient art of winemaking shape the *mental terroir* that will also determine the successful implementation of current and future wine yeast innovations. In this sense, this article also emphasises the reality of the adage that if the *past* could be changed, it would not exist; if the *future* could be stopped, it would not survive; and if the *present* could be avoided, it would not prevail. Therefore, today's wine stakeholders must be wise enough to learn from the past, smart enough to utilise the present and imaginative enough to anticipate the future with realism and optimism.

### Respect the past

Hindsight is often ridiculed as *belated perfect sight* based on *knew-it-all-along* misgivings. However, an in-depth, retrospective assessment of the failings and successes of past practices and traditions can facilitate the reinvention of traditions and the creation of new traditions. Relying on knowledge from the past does not have to restrict innovation through unnecessary inflexibility and conservatism. Nor should it reduce the wine industry's capability to successfully innovate to meet the ever-shifting demands for cost-effective production of wine with minimised resource inputs, improved quality and low environmental impact (Pretorius and Høj 2005).

Progressive thought-leaders will never dismiss the past in order to open doors to the future of winemaking. Leading researchers in viticulture and oenology, vintners and marketeers recognise the potential benefits of exploring past endeavours to develop innovative practices and products. The evolving practices over several millennia to produce wine that consumers want to drink are supported with guidance from the rich history of wine. It would therefore be counterproductive to downplay the role of the past in wine innovation. Integrating knowledge from the past into improved practices and products can elicit favourable market responses and legitimise innovations. The wine industry is in a privileged position to leverage knowledge from the past, its legacies and traditions, to keep innovating.

Some traditional grapegrowing and winemaking practices stem from historical narratives that maintain links to a specific wine-producing region's past. Ancestor symbolisation that calls to mind a wine region's past can nurture a strong socio-emotional attachment to such a territory's traditions. This often leads to the establishment of legacy councils or governing bodies responsible for designing policies and initiatives aimed at preserving a particular region's past, including its cultural heritage, reputation and tradition to provide a competitive advantage in the global wine market. This is the origin of *regionality* and the concept of *terroir* − a quint-essential French term (derived from the word *terre* or from the Latin word *terra*, meaning *earth, soil, terrain* or *land*) with no precise English equivalent (Robinson 1999). The International Organisation of Vine and Wine (OIV; www.oiv.int) defines ‘vitivinicultural *terroir* as a concept that refers to an area in which collective knowledge of the interactions between the identifiable physical and biological environment and applied vitivinicultural practices develops, providing distinctive characteristics for the products originating from this area. *Terroir* includes specific soil, topography, climate, landscape characteristics and biodiversity features’.

The term *terroir* is one of the most controversial words in the vocabulary of wine, and it is also one of the most used and least understood (Lewin 2010). *Terroir* is generally agreed to be the expression of the vineyard in a wine, but it has become erroneously synonymous with the view that the highest quality wine results spontaneously and purely from the ‘right’ locations. Where the concept of *terroir* is misleading is that it concludes that little intervention by the winemaker is required. It therefore comes as no surprise that this ill-defined and misrepresented cliché is the single most polarising reason for endless arguments in wine circles − from vineyards, wineries and wine cellars to boardrooms, newsrooms, retail outlets and restaurants. The fervour surrounding what constitutes *terroir* and the importance thereof is intense, and at times, irrational. On one extreme side of this debate, the belief that *terroir-is-the-be-all-and-end-all* is harnessed to justify the *naked-as-nature-intended* ideology in winemaking. On the other far end of the spectrum, the doubters who disparage the whole concept refer to *terroir* as hokum proffered by traditionalists desperately clinging to their waning share of the global wine market. It is safe to say that the truth lies somewhere between these two extreme interpretations of *terroir*.

**Figure 2.**
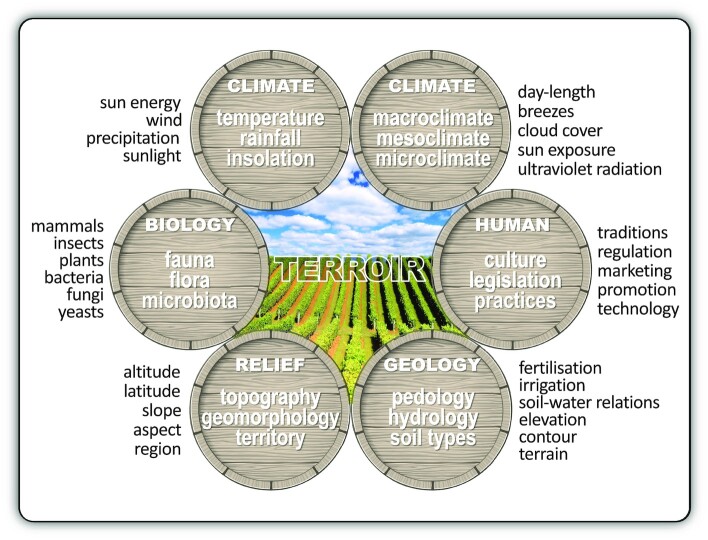
The concept of *terroir* in wine production. *Terroir* entails the total natural environment for specific viticultural sites, including climate as measured by temperature and rainfall; sunlight energy (or insolation) received per unit of land surface area; relief (topography or geomorphology) comprising altitude, slope and aspect; geology and pedology, determining the basic physical and chemical characteristics of various soil types; and hydrology or soil-water relations.

The notion that consumers can taste the earth in wine − for example wine ‘minerality’ stemming from vineyards planted in soft limestone soils − is tantalising but misleading. However, *terroir* does provide a welcome connection to nature and a specific locality in a globalised and increasingly delocalised world (Iland *et al*. 2009). *Terroir* gives wine a specific address − wines from *somewhere* rather than *anywhere*. For many, this sense of place − this sense of origin and authenticity − embodies the ultimate meaning of wine quality, whereas for others, it is a clichéd marketing tool with roots in hazy pseudoscience. Irrespective of one's own stance on this matter, *terroir* gained, over many centuries of winemaking, broad application and lies at the heart of the regulated demarcation of quality-hierarchies of wine-producing regions, such as the French wine *appellation d'origine contrôlée* (AOC) system and all the variations thereof in other countries. Such wine appellation systems presume that the land from which the grapes are grown imparts unique and recognisable region-specific qualities that cannot be replicated elsewhere in the world. In this sense, *terroir* describes the total natural environment for specific viticultural sites (Robinson 1999). This includes climate as measured by temperature and rainfall; sunlight energy (or insolation) received per unit of land surface area; relief (topography or geomorphology) comprising altitude, slope and aspect; geology and pedology, determining the basic physical and chemical characteristics of various soil types; and hydrology or soil-water relations (Fig. 2).

In the spirit of *respect for the past*, it is important to point out that long before the implementation of regulated appellation systems like the AOC in France, the concept of different grapegrowing regions having the potential to produce distinct wines with unique flavour profiles was established in the ancient world of winemaking. There is ample historical evidence that ancient Sumerian, Syrian, Phoenician, Greek and Roman civilizations codified certain places as important vineyard locations (McGovern *et al*. 2004; Iland *et al*. 2009; Chambers and Pretorius 2010). For example, in Ancient Greece, amphorae were stamped with the seal of the region where the grapes were harvested from, and soon different regions established reputations based on the quality of their wines. Ever since, the idea to label wine based on their origin − with certain vineyards and grapegrowing regions revered because of the high quality of wine they produced − grew and evolved over the millennia and eventually became part and parcel of winemaking practices and culture. Nowhere has the early concept of *terroir* expressed itself so prominently as in Burgundy where the monks of the Benedictine and Cistercian orders were able to cultivate grapes on vast landholdings and conduct large-scale observation of the influences that various parcels of land had on the quality of the wine they produced. Over the centuries, these recorded observations began to draw the boundaries of different *terroirs* along the contours of the Côte d'Or limestone escarpment and laid the foundations of today's Burgundian *Grand Cru* vineyards.

Nowadays, *terroir* is used to define virtually every wine-growing region in both the Old World and the New World, and has, to some extent, lost its meaning. In many instances geographical indication (GI) boundaries are now drawn by court orders after protracted legal battles and some of these boundaries are straight lines with no relationship to the clay, silt, loam, chalky, sandy or rocky soil patterns of those regions. No wonder the validity of the concept of *terroir* is, after centuries of trial-and-error experimentation with vine plantings the world over, called into question. There are those who say that the ambiguous term *terroir* is conveniently invoked as a marketing stunt to deceivingly link regional chemical composition to the sensory palate of consumers in a desperate attempt to find a point of distinction for their wine in an over-crowded marketplace. So, how can we bring scientific rigour and logic back into this increasingly sharp debate about *terroir* and future directions of contemporary winemaking? Clear evidence and scientifically sound data based on rigorous research is the only way to bust the myths of dupes and counteract the scepticism of doubters.

It does not require rocket science to presume that a vine of a particular variety grown in sunny, well-drained conditions at the top of a hill will produce dissimilar grapes from a vine of the same variety in shady, water-drenched conditions at the bottom of the hill. The holistic combination of all the natural elements of a vineyard site − the macroclimate, mesoclimate and microclimate as determined by latitude, elevation, contour, sun exposure and rainfall, as well as soil type, region-specific fauna and flora, including the microbiome present in vineyards − give each site its own *terroir* (Lewin 2010). These contributors might be reflected in the wines produced more or less consistently across vintages, to some degree irrespective of variations in viticultural and oenological practices. In other words, *terroir* encompasses all the environmental conditions that influence the biology of a vine in a particular viticultural site and thus the composition of the grape itself − nothing more and nothing less. This is generally not a point of contention. What is contested, however, is the degree to which *terroir* effects are unique, recognisable and commercially significant.

Opinions diverge greatly on the reality and, if real, the importance of *terroir* in determining wine qualities. Newer wine-producing regions might be missing out on the extraordinary prestige value and marketing power of the top-tier older vineyards backed-up by many centuries of practical trial-and-error matching and evolution of *Vitis vinifera* grape cultivars in viticultural sites, each with its own environmental peculiarities. However, vintners in these younger grapegrowing regions − unburdened by the rigidity and constraints of a highly regulated appellation system − argue that modern improvements in vineyard and winery technology helped raise and unify standards of wine quality, thereby obscuring differences in both style and quality of wines that in the past were (sometimes wrongly) attributed to *terroir* in its true sense (Iland *et al*. 2009). Paradoxically, these same technological advances can also serve to unmask the authentic differences due to *terroir*.

Unsurprisingly, there are those who wish to expand the definition of *terroir* to include elements that are controlled or influenced by human interventions and other cultural aspects. Human controlled aspects of *terroir* include vineyard management decisions, such as choice of grape variety, vine trellising systems, canopy management, controlled irrigation, fertilisation, herbicide application and yeast inoculation to name but a few. These agronomic practices do not only affect the biology of the vines and composition of the grape berries, they also impact the microbiome, which in turn, plays a critical role in fruit development, and consequently, might influence grape and wine quality properties. This has given rise to the concept of *microbial terroirs* − including *yeast terroirs* for that matter − for wine grapes (Gilbert, van der Lelie and Zarraonaindia 2014; Knight *et al*. 2015; Belda *et al*. 2017b).

In recent years, there has been a surge in the search for regional *microbial signatures* and microbial *biogeographies* of wine grapes, and the impact of the farming system and grape variety on bacterial, fungal and yeast communities in vineyards (Cordero-Bueso *et al*. 2011a; Canfora *et al*. 2018; Morrison-Whittle Lee and Goddard 2017). This is how wine scientists can demonstrate their *respect for the past* by taking the lead in the *terroir* debate with rigorous research and robust data. Research and data will aim to debunk some of the myths and clarify unequivocally what the realties are of, say, the influence of the region-specific microbiomes, and in fact, the *yeastomes* of grapes grown in niche *terroirs*. The time is ripe for a concerted effort from international consortia consisting of leading research groups to undertake such investigations at a grand-scale.

### Lead the present

In today's fast-paced digitised world in which the future seems to be approaching us faster than ever and arriving unannounced, vintners are confronted by the tension between the *stability* afforded by traditions and the *adaptability* demanded by innovations that uncork new opportunities. Today's wine innovators need to be wise *custodians* of the industry's past, open-minded *curators* of its rich traditions, innovative *stewards* of the here-and-now realities of an industry with a chronic structural oversupply of wine, and foresightful *architects* of a brighter future, all at once.

Currently, the global wine industry's main imperative is two-fold: ensure that the world's ∼8 million hectares of manicured vineyards are financially and environmentally sustainable; and that the ∼30 billion litres of wine that are produced annually are saleable despite ever-changing environmental conditions, dynamic consumer preferences and technological transformations (Pretorius and Høj 2005). To effectively address a chronic oversupply of wine, contemporary wine producers realise that today's *best* might not meet tomorrow's consumer preferences. However, this will require a paradigm shift and open mindset. To paraphrase Albert Einstein, we cannot solve our oversupply problems with the same thinking we used when we created them. So, despite the abundance of, and affection for, traditional methods of grapegrowing and winemaking, the industry in its totality will have no alternative than to follow the path of technological progress because that is the only way how the industry will be able to step up to changing PESTLE demands of the future. These demands include a complex blend of *political* (e.g. East-West power dynamics), *economic* (e.g. trade wars), *societal* (e.g. health concerns), *technological* (e.g. artificial intelligence, automated robotics, quantum computing and social media), *legal* (anti-alcohol regulation) and *environmental* (e.g. climate change) challenges.

Although the scope of this paper focusses on the pursuit of technological innovation involving yeasts − a small subset of these interconnecting PESTLE factors − the intent is not to trivialise the non-technological aspects; rather the intent is to consider current technological advances in our understanding of wine yeast biology and emerging technologies in full recognition of shifting political, economic, societal and environmental futures. There are, of course, also several non-yeast related technological advances in grapegrowing and winemaking. For example, as yeast biologists are opening new scientific frontiers and opportunities, so are scientists researching the fundamentals of grapevine varieties and wine-related bacteria.

Over the past 25 years or so, there was a deluge of new fundamental discoveries and further research opportunities relating to the biology of grapevine cultivars, malolactic bacteria and wine yeasts. For example, today we have access to the full genome sequences of many *Saccharomyces cerevisiae* strains (Goffeau, Barrell and Bussey 1996; Oliver 1996) and several other *Saccharomyces* (Borneman et al. 2008, 2011, 2012; Borneman and Pretorius 2015; Dunn *et al*. 2012; Novo *et al*. 2009; Peter *et al*. 2018) and non-*Saccharomyces* yeasts [e.g. *Hanseniaspora guilliermondii* (Seixas *et al*. 2016); *Torulaspora delbrueckii* (Tondini *et al*. 2018); *Hanseniaspora vineae* (Giorello *et al*. 2019)]; malolactic bacteria, including *Oenoccocus oeni* (Mills *et al*. 2005); and *V. vinifera* noble grape cultivars, such as Syrah, Cabernet Sauvignon, Chardonnay, Carménère, Nebbiolo and Tannat (Gambino *et al*. 2017; Minio et al. 2017, 2019; Roach *et al*. 2018). A multitude of population sequencing efforts revealed clonal diversity amid noble grape cultivars and strain-significant variation amongst malolactic bacteria and wine yeasts (Borneman et al. 2008, 2011, 2012; Hyma *et al*. 2011; Curtin *et al*. 2012; Borneman, Pretorius and Chambers 2013a; Borneman, Schmidt and Pretorius 2013b; Almeida *et al*. 2015; Marsit and Dequin 2015; Legras *et al*. 2018; Steensels *et al*. 2019;). These DNA-*reading* resources empower us to decode, unravel and delve into the molecular intricacies of grape cultivar and microbial strain differences that determine phenotypic traits, which impact fruit and wine quality.

These open-source data sets and knowledge are powerful decision-making tools in terms of choice of new grape planting material, viticultural practices in existing vineyards, selected microbial strains, fermentation conditions and other oenological processes. As an example, the comparative genome sequencing data for *S. cerevisiae* have enabled systems-based approaches aimed at the identification of gene targets that could improve the flavour profile of low-ethanol wine yeast strains (Varela *et al*. 2018). Data generated from such Systems Biology approaches are fundamental to contemporary yeast strain development programmes (including yeast clonal selection, breeding, mutagenesis, genetic and metabolic engineering), which, in turn, play an important role in assisting winemakers in their endeavour to produce low-alcohol wines with desirable flavour profiles (Pretorius 2000, 2016, 2017a; Goold *et al*. 2017b).

If yeast-strain developers take full advantage of the aforementioned DNA-*reading* technologies (coupled to molecular genetic systems approaches) in conjunction with the emerging DNA-*writing* and DNA-*editing* technologies (synthetic genomics), it is highly likely that the next generation of wine yeast innovations would come from Synthetic Biology. As *genetic engineering* approaches are now transitioning into *genome engineering* paradigms, the design, development and testing of customised wine yeast strains are bound to become more precise (Pretorius 2017a,b). Such yeast *avatars* will undoubtedly enlighten some of the yet-to-be discovered oenological secrets of what exactly makes a wine yeast tick under winemaking conditions and what differentiates one strain from another in terms of robustness, fermentation performance and flavour activity. The time is now for us to consider how to optimally utilise *avatar* prototypes as study models without alienating a tradition-conscious industry that would deny future applications of customised yeast strains.

### Secure the future

The wine industry's past is filled with invaluable *lessons*, the present with inspiring *opportunity* and the future with daunting *uncertainty*. What is certain, however, is that while the relative distance between the present and past increases, the distance to the future recedes at a blistering pace. To demonstrate foresight in anticipating and securing the future of the wine industry, leading minds in science often engage in mental *time-travel* by imagining, exploring, testing, questioning and debating future scenarios. For some, this is *science fiction* material for novels and films and for others it is a logical way to establish *scientific fact* through imaginative research, impactful discovery and innovation.

Epic science fiction (*sci-fi*) films, such as *Avatar*, imagine future scientific or technological advances and major social or environmental changes, often reaching for the stars by envisaging *multi-planetary species* living in robotic villages in space or colonising fictional celestial bodies, moons and planets. In this *geeky* genre, the plot frequently portrays heroic extraterrestrial beings or scientists trying to find innovative solutions to avoid dystopian futures. *Avatar* is no exception in this regard. In this film, scientists use artificial hybrid sapient humanoids called *avatars*. These *avatars* are wirelessly operated by genetically matched humans to explore the biosphere of the fictional alien Pandora moon orbiting a Saturn-sized gas giant, Polyphemus in the real Alpha Centauri system.

Well-researched and ingenious science fiction films encourage us to stand on the bridge between fantasy and reality and envision both positive futures and extreme futures of collapse. Science fiction gives us license to freely explore the boundaries of our own imagination and think about the unthinkable. It frees us up to imagine future worlds and question the *big issues* without any of the constraints that exist in the present moment. In so doing, *sci-fi* showcases our greatest fears and hopes for what might be possible and allows us to question where the world might be heading. The value of science fiction is that this genre often provides the first level of alert about things to come − provocative *prototypes* that engage and encourage people to envision and raise *inconvenient questions* about the direction of future technologies and social systems. In today's *tech-heavy* world that continuously plunges headlong into unknown futures, it is essential to regularly stand back to ponder the *big questions* and to analyse the potential impacts on people and planet.

In our 21st Century futurist world, science fiction is not quirky anymore; there are ample examples where projections in fanciful *sci-fi* novels and films eventually manifested in reality. There is often an undertone of realism in science fiction. One can therefore argue that there is a symbiotic relationship between pioneering researchers advancing the frontiers of science with their novel ideas and inventive technologies, and imaginative *sci-fi* storytellers with their creative magic. Reality often traverses the mystical boundaries between the worlds of *sci-fi* writers and film directors and those of scientists, inventors, innovators and futurists. This does not suggest that every fictional concept will eventuate as something concrete in real life; however, the co-dependency between science fiction and science fact stimulates thought-provoking *what-if?* questions and help us to expect the unexpected.

These *what-if?* scenarios frequently inspire public discourse about the advantages and disadvantages, opportunities and challenges, risks and safeguards of emerging sciences and *avant-guard* technologies such as Synthetic Biology (colloquially referred to as *synbio*) and the capability to *read*, *write* and *edit* DNA codes of genes, chromosomes and genomes of various lifeforms. Inspired by the progress of the international *Synthetic Yeast Genome* project (known as *Yeast 2.0* or *Sc2.0*), this article asks, amongst other things, *what if* synthetic genomics intersects with the ancient art of winemaking? What lies beyond the completion of the *Yeast 2.0* project (Pretorius and Boeke 2018) and the reality of a physiologically fit laboratory strain of *S. cerevisiae* powered by 16 chemically-synthesised chromosomes? If we are to direct an unscripted futuristic *sci-fi* film about *synbio* today, what are the known-knowns, the known-unknowns and the unknowns-unknowns? *What if* familiar things play out in unfamiliar ways? *What if* future incarnations of *wine yeast avatars* come along and disrupt the traditions of one of the world's oldest biotechnological processes that ‘magically’ turns grapes into flavoursome wines with preservative properties and hedonic and psychotropic effects? *What if* the *alien forces* of innovators invade the *terroir* of traditionalists and uncloak wine from its mystique and romanticism?

By asking these *what if?* questions, we can peek beyond the horizon and synthesise a link between *what we know now* and *what could be*. To help secure the future of wine, yeast biologists are tasked to build a bridge of data and knowledge between science fiction-like experiments in today's leading laboratories and tomorrow's vineyards and wineries.

## STAMPING YEAST SIGNATURES ONTO THE TERROIR AND VINTAGE LABELS OF WINE

*Bottled poetry* is a delicious phrase that often rolls off the tongue of wine connoisseurs when they refer to *fine* wine. In the context of this metaphor, grapes and yeasts are the *ink* and *pen* with which the *poets* − viticulturists and oenologists − co-write *fine wine poetry* on the *terroir sheets* of their vineyards and the vintages of their wine. When these two natural *ink-and-pen* companions combine, a complex mixture of grape- and yeast-derived (and in some cases bacterial and oak-derived) compounds emerge, which largely define a wine's appearance, aroma, flavour and mouth-feel properties (Swiegers et al. 2005, 2007a,b; van Wyk, Kroukamp and Pretorius 2018). The grape-derived compounds provide varietal distinction in addition to giving wine its basic structure while yeast fermentation gives wine its vinous character. Wine attributes are the result of an almost infinite number of variations in production, whether in the vineyard or the winery.

In *spontaneous* fermentations − uninoculated ferments − there is a progressive growth pattern of indigenous yeasts (also referred to as *autochthonous*, *natural*, *wild* or *feral* yeasts) with the final stages invariably being dominated by the Crabtree-positive, alcohol-tolerant strains of *S. cerevisiae* (universally known as *the wine yeast*). The primary role of wine yeast is to catalyse the rapid, complete and efficient conversion of grape sugars (glucose and fructose) to ethanol, carbon dioxide and other minor, but important flavour-active metabolites (e.g. acids, alcohols, carbonyls, esters, terpenes and thiols), without the development of off-flavours (e.g. hydrogen sulfide) (Lilly *et al*. 2006; Swiegers *et al*. 2005; Cordente et al. 2009, 2012, 2013). To achieve this outcome, a Crabtree-positive carbon metabolism is the most efficient strategy for grape sugar utilisation (with a preference for glucose over fructose) that maximises ethanol production. This adaptation in *Saccharomyces* enables energy generation under fermentative or anaerobic conditions and limits the growth of competing microbes − including non-*Saccharomyces* yeasts − by producing toxic metabolites, such as ethanol and carbon dioxide. Therefore, *S. cerevisiae*’s effective *make-accumulate-tolerate-consume* alcohol strategy makes it the preferred yeast species for initiating wine fermentations in *inoculated* (or *guided*) ferments (Goold *et al*. 2017).

**Figure 3.**
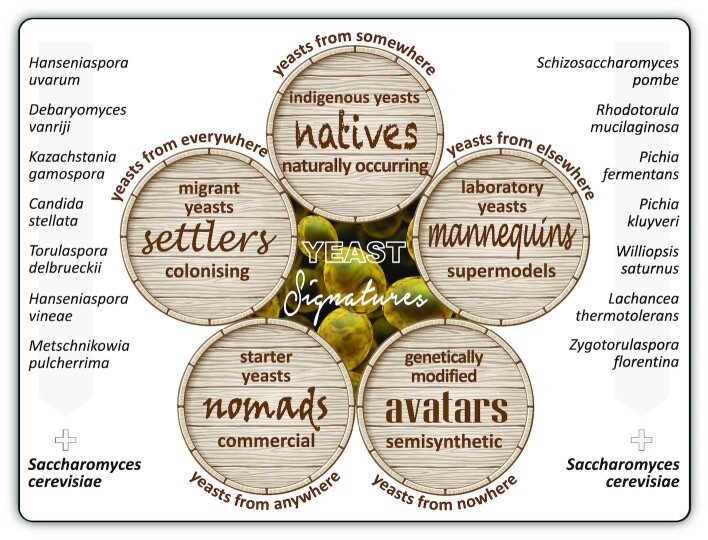
Yeast imprints on the sensory profile of win**e**. A diverse range of flavour-active yeasts can contribute to the overall quality of a wine. Flavour-active yeast types that might constitute a specific *yeastome* relevant to wine quality can include the following kinds of yeasts: The indigenous *Saccharomyces* and non-*Saccharomyces* yeasts inhabiting a niche vineyard are referred to as *natives* and yeasts that migrated and accumulated in a winery are referred to as *settlers*. Commercial active-dried yeast starter strains used in many wineries across the world are referred to as *nomads*. Laboratory-bred strains of *S. cerevisiae* are termed supermodel *mannequins* while genetically engineered and semisynthetic yeasts with reinvented or edited genomes are called *avatars*. The following sections explore the importance of residential *natives* (indigenous yeasts from *somewhere*), colonising *settlers* (migrant yeasts from *everywhere*), imported *nomads* (commercial starter yeasts from *anywhere*), prototypical *mannequins* (quintessential model yeasts from *elsewhere*) and alien *avatars* (genetically modified yeasts from *nowhere*) in terms of our understanding of their role in current wine ferments or potential role in future winemaking practices.

Grape must is not sterile and naturally contains, amongst other microbes, a mixture of *Saccharomyces* and non-*Saccharomyces* yeast species; therefore, wine fermentation is not a *single-species* fermentation process. The indigenous non-*Saccharomyces* yeasts, often already present in the must at much greater numbers than *S. cerevisiae*, are adapted to the specific environment and in an active growth state, which gives them a competitive edge. However, the eventual dominance of *S. cerevisiae* in both spontaneous and inoculated ferments is essential to ferment wine to dryness (∼1-2 g/l of residual sugar). The length of time during which the non-*Saccharomyces* and non-*cerevisiae Saccharomyces* yeasts are allowed to participate in these *multi-species* ferments is the choice of the winemaker. More than 40 of the 1500 known yeast species have been isolated from grape musts (Jolly *et al*. 2014). Some of these wine-related non-*Saccharomyces* yeasts (e.g. *Brettanomyces bruxellensis*) cause off-flavours (e.g. volatile acidity and phenolic odours) while others contribute positively to a wine's aromatic *complexity* and *textural roundness*. For example, if a winemaker deems that the risk of high concentrations of *vinegary* volatile acidity or *leathery* ethylphenols outweighs the beneficial metabolites of non-*Saccharomyces* yeasts in a particular grape must, the pH of the fermenting grape juice can be lowered and a higher dosage sulfite can be added to restrict the feral spoilage yeasts, thereby allowing the starter culture strain of *S. cerevisiae* with which the ferment was inoculated to gain dominance faster. It is generally accepted that risk management with *spontaneous* (*multi-species*) ferments is more complex than with *guided* (mostly inoculated with a single species) ferments. To gain the benefits of both practices without risking negative effects of spoilage yeasts, some winemakers prefer to inoculate their grape musts with selected non-*cerevisiae* and/or non-*Saccharomyces* yeast strains along with one of more of the > 250 different commercially-available wine strains of *S. cerevisiae* (Fig. 3).

Over the millennia, thousands of clonal varieties of *V. vinifera* vines − originating from Transcaucasia − evolved and spread across the world while vintners matched their traits to the *terroir* of each newly-planted vineyard site and the likings of their targeted consumers (Vivier and Pretorius 2000, 2002). At the same time, the commensal microbial flora that coexisted with the vines in those new vineyards similarly evolved. However, to date, the *microbiome* of wine-producing regions and the potential influence of *microbial terroirs* on wine quality have not received the same level of scientific scrutiny (Gilbert, van der Lelie and Zarraonaindia 2014). Investigation of the importance of yeast communities associated with the grapes from a particular vineyard or region is complex because some ambient yeasts might originate from a neighbouring vineyard or be imported via oak barrels and commercial yeast starter cultures used in a nearby winery (Knight *et al*. 2015). Conditions and practices applied in both the vineyard and winery can dramatically alter the composition of grape and wine-related *yeastomes* in a particular setting (Bokulich *et al*. 2013a,b; Setati *et al*. 2012, 2016).

For clarity, the following nomenclature is used here to differentiate flavour-active yeast types that might constitute a specific *yeastome* relevant to wine quality. The indigenous *Saccharomyces* and non-*Saccharomyces* yeasts inhabiting a niche vineyard are referred to as *natives* and yeasts that migrated and accumulated in a winery are referred to as *settlers*. Commercial active-dried yeast starter strains used in many wineries across the world are referred to as *nomads*. Laboratory-bred strains of *S. cerevisiae* are termed supermodel *mannequins* while genetically engineered and semisynthetic yeasts with reinvented or edited genomes are called *avatars*. The following sections explore the importance of residential *natives* (indigenous yeasts from *somewhere*), colonising *settlers* (migrant yeasts from *everywhere*), imported *nomads* (commercial starter yeasts from *anywhere*), prototypical *mannequins* (quintessential model yeasts from *elsewhere*) and alien *avatars* (genetically-modified yeasts from *nowhere*) in terms of our understanding of their role in current wine ferments or potential role in future winemaking practices (Fig. 3).

### Yeasts from somewhere

*Native yeasts* residing in a niche site represent an important component of the *microbiome* of a vineyard. Across viticultural zones and over time, the microbiota (e.g. acetic acid bacteria, botrytis fungi and yeasts) that inhabited a vineyard and colonised the phyllosphere of *V. vinifera* can substantially affect grapevine health, fruit development and ripening, as well as the quality of grapes and wine (Gilbert, van der Lelie and Zarraonaindia 2014). There is mounting evidence that the non-random biogeographical distribution patterns of microbial assemblages of grape surface microbiota in vineyards can be modulated by a combination of several factors. These factors include geographical location, farming system, soil, cultivar, vintage and climate to varying degrees. However, not all members of a vineyard's microbiome can complete the *from-vine-to-wine* journey because many of them cannot withstand the low-pH, high-ethanolic and anaerobic conditions of wine fermentations (Bokulich *et al*. 2013a,b, 2016). Nevertheless, there is renewed interest to establish an indisputable link between differential *geographic phenotypes* and *sensorial signatures* as encapsulated by the concept of *terroir*. The key hypothesis of such investigations is that non-random yeast inhabitants with specific *vineyard postcodes* form part of the *microbial terroir* of grapes harvested from well-established vineyards and can influence the chemical and sensorial profile and the so-called *typicity* of the wine in a unique, reproducible and recognisable manner (Belda *et al*. 2017b).

The microbiome of a grapevine plant has direct and indirect relationships with its host (Gilbert, van der Lelie and Zarraonaindia 2014). For instance, these relationships are affected by the availability of organic matter and essential nutrients in the soil (including nitrogen fixation) and environmental stresses (e.g. water stress caused by drought or stresses caused by the presence of phytotoxic contaminants). Other factors that play a role include the degree of phytopathogens activity in terms of competition for space and nutrients, antibiosis, production of inhibitory enzymes (e.g. hydrolytic enzymes) and systemic induction of plant defence mechanisms. Soil endophytes in the rhizosphere of the vine and those that migrate through the plant to colonise aerial tissues internally or externally (epiphytes) can, for example, have several metabolic activities that support vine health by either promoting the physiology or suppressing disease-causing pathogens, which, in turn, can alter the microbial composition that end up in grape must (Gilbert, van der Lelie and Zarraonaindia 2014).

It would seem logical to assume that the same physical and chemical criteria that determine which vines grow well in conditions prevailing in certain sites (e.g. soil nutrients levels, solar radiation, temperature, humidity and precipitation) would also impact the biography of microorganisms in a vineyard's ecosystem (Gilbert, van der Lelie and Zarraonaindia 2014). To understand the biogeographical regionalisation of microbial communities of site-specific vineyards and regions, it would also be necessary to determine if the grapevine plants themselves select for different microbiota based on their physiological response to different environmental conditions and viticultural practices.

This century has seen a marked increase in sophistication of technologies with which the microbiome of a vineyard can be investigated. Recent studies are starting to shed some light on how farming practices (e.g. soil cultivation, fertilisation, irrigation and the application of herbicides, pesticides and fungicides) and some oenological practices in wineries (repeated use of selective yeast starter cultures) are shaping the composition of the microbiome of vineyards (Cordero-Bueso *et al*. 2011b; Belda *et al*. 2017b; Canfora *et al*. 2018; Morrison-Whittle, Lee and Goddard 2017; de Celis *et al*. 2019). For example, by using next-generation sequencing of 16S rRNA and internal transcribed spacer (ITS) sequences of ribosomal DNA to determine the relative abundances of bacteria, mycelial fungi and yeasts, it has become clear that vineyard under-vine floor management alters the microbial composition of soil but does not seem to affect any shifts in the fruit-associated microbiome (Chou *et al*. 2018). However, other vineyard management practices and environmental factors are more influential in shaping not only the grape-associated microbiome, but also its later behaviour in wine fermentation (Grangeteau *et al*. 2017).

By using a high-throughput, short-amplicon sequencing approach, researchers were able to demonstrate that regional and site-specific factors along with grape variety-specific factors shape the fungal and bacterial consortia inhabiting the surfaces of grape berries (Gilbert, van der Lelie and Zarraonaindia 2014). These communities were shown to be correlated to specific climatic features, thereby demonstrating a link between environmental conditions and microbial inhabitation patterns in vineyards. It was shown that the degree of differentiation among these grape-surface microbial communities from different regions were substantially increased when the biogeography was investigated within a grape variety of a particular vintage (Bokulich *et al*. 2013a,b, 2016). It was found that the host genotype, and therefore the phenotype of the grape cultivar, along with local and interannual (seasonal) climate variation (vintage) play a significant role in determining the nature of the microbial assemblages on the surface of wine grapes. The authors of this study suggested that these factors appear to shape the unique inputs to regional wine fermentations. They further proposed the non-random existence of *microbial terroir* as a determining factor among grapes harvested from different grape varieties and from regions with different climatic conditions.

### Yeasts from everywhere

Yeast *settlers* can colonise a wide variety of natural and man-made habitats, including vineyards and wineries, to form microbial communities associated with specialised niches, such as vineyard soils, grapevine plants, grape skins and the surfaces of equipment used within wineries. Gaining deeper insights into the composition, population dynamics, dispersal and maintenance of these yeast communities along their journey from the vineyard to the winery can potentially clarify the relations between the microbiomes associated with vine health, grape yield, grape and must quality, and the metabolome of wine impacting the sensorial profile of the end-product.

The composition of microbial communities associated with pre-crushed grapes − including the variety and quantity of yeast species − depend on factors, such as the method of harvest (hand-picked or mechanical), grape temperature (day or night harvest), grape condition (biotic and abiotic damage, degree of ripeness, grape variety), sulfite addition, and the time between harvesting and crushing of the grapes (distance and duration of transport from the vineyard to the winery, ambient temperature and initial grape temperature) (Gilbert, van der Lelie and Zarraonaindia 2014). The population profile of yeasts present in grape must can also be significantly influenced by the method and intensity of grape destemming and crushing (grape-stomping or mechanical crushing with various types of wine presses), cellar hygiene (sanitation protocols and disinfectants used), must pre-treatment (aeration, sulfite addition, enzyme treatment, clarification protocol, temperature) and inoculation with starter yeast cultures (Jolly *et al*. 2014).

Untreated grape must provides a rich nutritive niche for yeasts to grow. However, cellar hygiene practices, low pH conditions, high osmotic pressure, sulfite concentrations and temperature can make the grape pulp and winery environment much harsher for the less robust species (Jolly *et al*. 2014). These factors and the anaerobic conditions that sets in when fermentation commences are bound to stack the odds against bacteria and fungi with oxidative metabolisms. This is also true for yeast species and any other microbes that are more sensitive to high sugar levels (varying from 150 to 250 g/l in ripe grapes) and sulfite concentrations (free SO_2_ levels ranging between 25 and 30 mg/l) in conjunction with low pH levels (varying between pH 3.0 and 3.6), suboptimal fermentation temperatures (ranging from 12 to 18°C for white wines, and from 20 to 30°C for red wines) and high alcohol levels (12%-15%) toward the end of fermentation (Jolly *et al*. 2014).

It is reasonable to expect that microbes able to survive these harsh conditions could accumulate on the surfaces of large specialised equipment, oak barrels and other winery tools and surfaces. Wineries could therefore serve as reservoirs of resident microbial communities, which might shape the microbiota in wine fermentations and perhaps even vector wine spoilage organisms Bokulich *et al*. 2013a). Robust data on the degree that winery milieus influence the microbial profile in fermenting grape juice is relatively scant and not well understood at a systems level. However, there are some reports that indicate that winery surfaces harbour seasonally fluctuating microbial populations with site-specific dependencies shaped by technological practices, processing stage and season. During each vintage, grape- and fermentation-associated microbes populate most winery surfaces, serving as potential reservoirs for microbial transfer between fermentations (Bokulich *et al*. 2013a). Winery surfaces usually house a fair amount of alcohol-tolerant *S. cerevisiae* strains and other yeasts, which could potentially act as an important vector of these yeasts in wine fermentations. However, there is mounting evidence that resident microbial assemblages on winery surfaces, before and after harvest, comprise microorganisms with no known link to wine fermentations with almost no spoilage microbes, suggesting that winery surfaces do not overtly vector wine spoilage organisms under normal cleaning and operating conditions (Bokulich *et al*. 2013a).

Regardless of the umpteen variables in grape harvest and winery operating conditions, the yeast species generally found on grapes and in wines are similar everywhere in the world. However, the proportion or yeast population profile in various wine-producing regions projects distinct differences.

### Yeasts from anywhere

Yeast *nomads* can be imported from anywhere as commercial starter culture strains to take control of the fermentation process by outcompeting bacteria and fungi, as well as *native* and *settler* yeasts. The concept of inoculating grape must with a starter strain of *S. cerevisiae* originated in the late 1800 s but was based on discoveries that dates back to the late 1600 s. It all started in the 1670 s when Antonie van Leeuwenhoek observed and described microscopically-tiny creatures for the first time and Louis Pasteur, in the 1850 s, proved that these sub-visible entities were living yeast cells responsible for fermentation. When Emil Christian Hansen succeeded in isolating the first pure yeast culture, Julius Wortmann and Herman Müller-Thurgau were quick to introduce the concept of inoculating wine ferments with pure yeast cultures in the 1890s. But it was not until 1965 that this innovation was taken a step further when the first pure culture of a *S. cerevisiae* wine yeast strain from Red Star became commercially available to winemakers in California. Since then, the commercial production of yeast used in the food and fermented beverage industries exceeds 1.8 million tons per year (Joseph and Bachhawat 2014). It is estimated that there are approximately 250 commercial strains available to the global wine industry as *active dry yeast* (ADY) cultures.

Despite the availability and abundance of *easy-to-use* commercial starter culture strains with proven desirable oenological characteristics, there is still an ongoing debate as to whether wine ferments should be allowed to occur by the action of grapevine-associated *native yeasts* and winery-residential *settler* yeasts or be driven by inoculated *nomadic* strains. However, this debate has now matured and transitioned from an *either-or* paradigm into a new paradigm with multiple options, i.e. spontaneous ferments, single-species ferments inoculated with one or more strains of *S. cerevisiae*, and mixed-species ferments inoculated with one or more non-*Saccharomyces* yeasts alongside at least one robust wine strain of *S. cerevisiae* (Belda *et al*. 2017a; Jolly *et al*. 2014). These kinds of fermentation options are referred to as *multistarter*, *mixed-culture* and *co-culture* ferments, in which strains can either be *sequentially* or *simultaneously* inoculated. These decision options enable modern-day winemakers to choose whether to leave their uninoculated grape musts until fermentation commences spontaneously or to guide their wine fermentations by using *single-strain* or *multi-strain S. cerevisiae* inoculation strategies along with a mixture of selected non-*Saccharomyces* strains.

**Figure 4.**
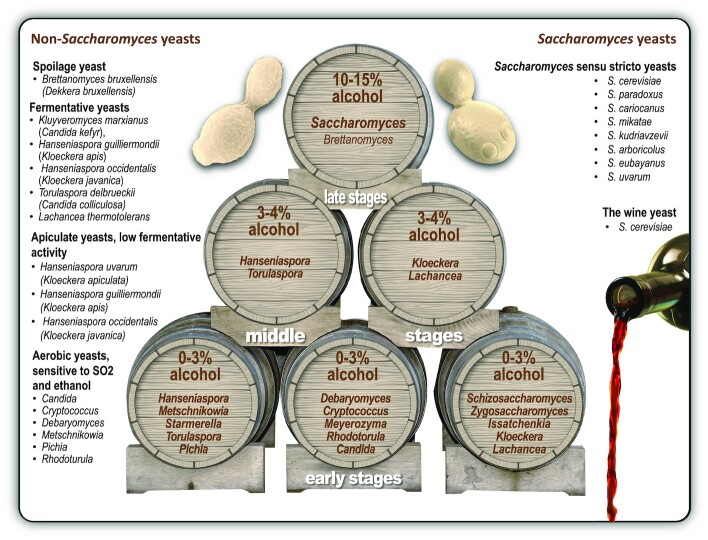
The sequential participation of various yeast species during wine fermentation. The selective pressures prevailing in fermenting grape must (e.g. sugar-induced osmotic pressure), fermentation conditions (e.g. temperature and pH) and winemaking practices (e.g. sulfite additions) inhibit and/or eliminate non-*Saccharomyces* yeast species during the course of fermentation. The selective nature of grape must becomes more pronounced once anaerobic conditions are established, nutrients become depleted and rising alcohol levels start to constrain the survival of ethanol-sensitive yeasts. This diagram is adapted from Pretorius (2017b).

Every winemaker knows that when crushed grapes or must remain in a vat, fermentation will commence spontaneously after a while. They also know that, during the initial phases of fermentation, non-*Saccharomyces* yeasts are both present and active in all ferments until an alcohol level of 3–4% is reached and before *S. cerevisiae* starts to dominate, whether inoculated or not (Fig. 4). The most sulfite- and alcohol-sensitive non-*Saccharomyces* yeast species will die-off at this point, but some of the more resilient species could remain metabolically active in later phases of the fermentation. There are three groups of these non-*Saccharomyces* yeasts (Jolly *et al*. 2014). The first group includes yeasts that are mostly aerobic, such as species of *Candida*, *Cryptococcus*, *Debaryomyces*, *Pichia* and *Rhodoturula*. The second group comprises apiculate yeasts with low fermentative activity, such as *Hanseniaspora uvarum* (*Kloeckera apiculata*), *Hanseniaspora guilliermondii* (*Kloeckera apis*) and *Hanseniaspora occidentalis* (*Kloeckera javanica*). The third group consists of yeasts with a fermentative metabolism like *Kluyveromyces marxianus* (*Candida kefyr*), *Metschnikowia pulcherrima* (*Candida pulcherrima*), *Torulaspora delbrueckii* (*Candida colliculosa*) and *Zygosaccharomyces bailii*.

The only non-*Saccharomyces* yeast that is able to remain metabolically active at the end of fermentation when the alcohol concentration reaches 12%–15%, is the spoilage yeast *Brettanomyces* (*Dekkera*). It is important to note that viable *Brettanomyces* cells are often not ‘culturable’ in the laboratory (Capozzi et al. 2016). In practice this means that, even in the presence of relatively high concentrations of SO_2_, *Brettanomyces* cells can remain dormant as opposed to being eliminated. Winemakers should therefore always be on guard to maintain appropriate SO_2_ levels during fermentation to avoid the ‘resuscitation’ of this spoilage yeast during wine ageing. The latter poses an ongoing challenge to the global wine industry in that strains of *B. bruxellensis*, which are more tolerant to higher levels of ethanol and sulfite than other non-*Saccharomyces* yeasts, can contaminate winery equipment, such as oak barrels, and, when they remain metabolically active post-fermentation, they could produce off-flavours. During wine maturation, particularly in oak barrels, *B. bruxellensis* can survive for prolonged periods of time and decarboxylate hydroxycinnamic acids to *p*-coumaric acid, ferulic acid and caffeic acid to their corresponding vinyl derivatives, which, in turn, can subsequently be reduced to 4-ethylphenol, 4-ethylguaiacol and 4-ethylcatechol. These compounds impart an undesirable *leathery*, *medicinal* or *metallic* aroma (Curtin *et al*. 2012; Curtin and Pretorius 2014). The most practical way to keep *B. bruxellensis* at bay is for winemakers to apply a strict hygiene regime in their wineries (especially oak barrels), to maintain appropriate concentrations of free SO_2_, and to inoculate the grape must with a fast-fermenting wine strain of *S. cerevisiae*. In other words, an effective way to safeguard wine against *Brettanomyces* spoilage is to conduct fermentation in clean wineries and under low-pH, high-sulfite, high-ethanol and anaerobic conditions that restrict *Brettanomyces* but still allows *S. cerevisiae* to prevail and dominate (Curtin and Pretorius 2014). However, this frontline in the battle against ‘Brett’ might change if *B. bruxellensis* strains evolve and become more resistant to standard sulfite dosages used in winemaking. That is why genomic insights into the evolution of *B. bruxellensis* has gained so much interest in recent times.

### Yeasts from elsewhere

*Saccharomyces cerevisiae* S288c is a laboratory-bred *mannequin* yeast with superstar status in the world of supermodels and breakthrough scientific discoveries. This *out-of-this-world* single-cell model eukaryote tops the league table of A-listed supermodel organisms. *S. cerevisiae* stands tall in Life Sciences’ *hall-of-fame* amongst other supermodel organisms, such as the bacterium *Escherichia coli*, the fruit fly *Drosophila melanogaster*, the nematode *Caenorhabditis elegans*, the plant *Arabidopsis thaliana* and the zebrafish, *Danio rerio*. Some of the most profound fundamental principles of biology were uncovered by using these supermodel organisms. These landmark discoveries include the definition of sub-cellular structures and the function of organelles; the basic principles of heredity, including the structure of genomes, chromosomes and genes, the genetic code, the rules of DNA replication and transcription, mRNA translation and protein synthesis; the regulation of genes and metabolic pathways; as well as the development of recombinant DNA technologies (e.g. gene cloning and transformation) and *in vitro* amplification, sequencing, editing and synthesis of DNA (Alfred and Baldwin 2015).

Just as the role of the fashion world's supermodels is to herald what the future wears, rather than accurately representing the ordinary version of *Homo sapiens* in the street, model organisms, such as *S. cerevisiae* S288c, are fashioned for experimental use in laboratories and not necessarily to be a mirror-image of other members of their own species or their nearest relatives living at the wild frontier of the real world. For example, *S. cerevisiae* S288c does not have the robustness and effervescent power to rise bread dough, brew beer or sparkle wine. However, building on the foundational milestone discoveries by Antonie van Leeuwenhoek (1676), Antoine-Laurent Lavoisier (1789), Joseph Gay-Lussac (1815), Friedrich Erxleben (1818), Charles Cagniard de la Tour, Friedrich Kützing, Theodor Schwann (1825), Julius Meyen (1837), Louis Pasteur (1857), Emil Hansen (1888), Eduard Buchner (1897), Øjvind Winge (1935) and Carl Lindegren (1943), strain S288c was specifically developed to control and manipulate its life cycle and genetics for research purposes. By several rounds of crossing selected parental strains and sporulation inductions, S288c was isolated as a strain that can be maintained as a stable haploid. Very early on, yeast researchers adopted S288c as the reference strain for studying the life and cell cycles of *S. cerevisiae*, genetic hybridisation, genetic engineering, genome sequencing and genome engineering. Today, S288c and its derivate haploid strains of both mating-types (a and α) are the mainstay yeast strains for genetic, genomic, transcriptomic, proteomic, metabolomic, fluxomic and interactomic research, to name but a few (Liti 2015).

The superstar status of *S. cerevisiae* is boosted by its proven safety track-record as a long-time domesticated, food-grade yeast, its compartmentalised sub-cellular structure (including an encapsulated nucleus) typical of a eukaryote, and the uncomplicated, inexpensive way of culturing it rapidly in the laboratory (Pretorius 2000, 2017a,b). Under optimal culturing and nutritional conditions, all three basic cell types (a, α and a/α) can double their mass every 90 minutes through an asexual mitotic budding process. Heterothallic a/α diploids lacking the *HO* mating-type switch gene, can be induced to undergo meiosis and sporulate, thereby generating stable haploids of both mating-types (*MAT*a and *MAT*α). In turn, *MAT*a and *MAT*α haploids can mate and give rise to *MAT*a/*MAT*α diploids, capable of sporulating and generating four new ascospores per ascus (tetrad), two of each mating-type. Homothallic haploids are less useful for classical genetic analyses because they can switch their mating-types from *MAT*a to *MAT*α and *vice versa*, and then self-mate. So, haploid ascospores derived from homothallic diploids can establish diploid lines (i) by mating with their own mitotic daughter cells after a mating-type switching event (*haploselfing*); (ii) by mating with another sibling ascospore stemming from the same meiotic event (intra-tetrad mating); or, more rarely and (iii) by mating with an unrelated individual (*outcrossing*) (Liti 2015). In homothallic haploids, the *HO* gene can utilise the information from the silent *HML* and *HMR* loci − located on either side of the *MAT* locus on Chromosome 3 − and dictate the switching between *MAT*a and *MAT*α. The *haplontic* phase of homothallic strains are therefore much shorter than the *diplontic* phase of their sexual life cycle. Both heterothallic and homothallic strains can asexually reproduce in the *MAT*a and *MAT*α haploid state or state of higher ploidy (from diploids to heptaploids) and aneuploidy (abnormal number of chromosomes per cell, i.e. not 16, 32, 48, etc). Most laboratory strains of *S. cerevisiae* are heterothallic haploid or diploid strains, whereas industrial wine strains can be either heterothallic or homothallic, and they are mostly diploid or aneuploid, and occasionally polyploid (Pretorius 2000, 2017). The ability to control the life cycle of *S. cerevisiae* and to switch it between mitotic and meiotic reproduction, and to develop strains with different ploidies surpass the experimental flexibility of any other model organism.

To fully understand what makes wine-related yeasts tick, it is important that we bridge the faultline between *cellular-molecular-developmental* research into the decontextualised *S. cerevisiae* S288c model strain and *ecological-evolutionary* research into the natural histories of both *Saccharomyces* and non-*Saccharomyces* yeasts in the wild (Liti 2015). For example, recent population genomics have illuminated the evolutionary history and natural genetic variations within subpopulations of *S. cerevisiae* (Hyma *et al*. 2011; Almeida *et al*. 2015; Goddard and Greig 2015; Marsit and Dequin 2015; Legras *et al*. 2018; Belda *et al*. 2019; Steensels *et al*. 2019). By studying its life cycle in natural settings with fluctuating environmental conditions, yeast ecologists and molecular biologists are closer to understanding the origin of *S. cerevisiae*. One of the natural habitats of *S. cerevisiae* is oak bark, which is subject to seasonal changes and cycles of in sap flow in *Quercus* oak trees. Cells of *S. cerevisiae* are therefore likely to spend most of their lifetime in a non-dividing (*quiescence*) state. Population genomic studies also indicated that budding yeast mostly reproduce asexually and that outcrossing (outbreeding) is rare in the wild but is not restricted to mating within a species (Liti 2015). Introgressed genomic regions and interspecies hybrids between *S. cerevisiae* and other members of the *Saccharomyces sensu stricto* complex can generate viable hybrids when interbred. For example, hybridisation between *S. cerevisiae* and *S. eubayanus* generated the hybrid species *S. pastorianus*, which is now widely used in the brewing industry (Liti 2015). Deliberate genetic breeding (crossing, spheroplast fusion and rare-mating) has also been successfully applied to generate superior bread, beer and wine strains of *S. cerevisiae*.

In addition to human-related environments, such as baking, brewing and winemaking, population genomics has also uncovered *S. cerevisiae* strains in primary forests in China that are remote from human activity, thereby indicating that this yeast species has a distribution more widespread than what was previously been postulated (Wang *et al*. 2012). This brings into question a long-held notion that *S. cerevisiae* is a *man-made organism* and queries whether it is only a coincidence that winemakers have favoured oak (*Quercus alba*, *Quercus petraea* and *Quercus robur*) as cooperage material for fermentation or maturation vessels.

Genetic variants within some lineages of *S. cerevisiae* have been shown to be nearly unique to subpopulations, such as European wine strains; Malaysian bertram palm-associated strains; strains from North American woodlands; Japanese saké strains; and West African strains associated with food and beverage fermentations. In some cases, phenotypic variation tends to follow population structure and some of these lineages are characteristic of domesticated breeds linked to distinct fermentation processes. In one sense, domesticated strains transcend geographic boundaries, share recent ancestry and reflect human migration history, including the transport of wine in oak barrels. In other cases, it is clear that some lineages are not linked to human activity; rather they are characteristic of specific geographic areas. For instance, the ancient and surprisingly divergent lineages within the well-structured population of Chinese isolates from primeval forests showed to be a remarkable reservoir of natural genetic variants for future investigations and potential applications.

Distinctions between model and non-model yeasts will become increasingly clear as yeast ecologists, organismal biologists and molecular geneticists apply metagenomic tools to their *broadband* mega field surveys of non-model yeast strains and their *laser-focussed* laboratory-based genomic analyses of *S. cerevisiae* model strains (Alfred and Baldwin 2015; Liti 2015). This does not imply that the in-depth molecular probing of a laboratory-bred strain like S288c should be slowed down. Its well-understood sexual and asexual reproduction cycles, along with the accessibility of its genetic system and the ease of gene transformation procedures make *S. cerevisiae* irreplaceable as a trailblazer for yeast research. This tractable supermodel yeast of GRAS status can rise to almost any challenge that contemporary science can pose. *S. cerevisiae* is amenable to nearly all types of genetic modification (breeding, mutagenesis and cloning) in the pursuit of probing the fundamental intricacies regarding molecular and cellular aspects of biology (Pretorius Curtin and Chambers 2012).

The versatility of *S. cerevisiae* as an unequaled eukaryotic supermodel organism in research laboratories and the most-used microbial workhorse in many fermentation industries is evidenced by several *world-firsts* (Pretorius 2017a). In ancient times, unknowingly and serendipitously, this yeast was the first microbe to be domesticated for the production of wine and other fermented products. It was also the first microorganism to be observed under a microscope and described as a living biochemical agent responsible for the transformation of sugar into alcohol and carbon dioxide. In modern times, *S. cerevisiae* became the first host organism for the production of a recombinant vaccine (against hepatitis B) and a recombinant food enzyme (the milk-coagulating enzyme, chymosin, for cheese making). Nowadays, *S. cerevisiae* is the most popular microbial cell factory for a range of products.

These practical applications are backed-up by an extensive, online searchable database (www.yeastgenome.org) and research tools. The S288c strain became the first eukaryote to have its whole genome sequenced and from there a full set of libraries of gene deletions, overexpression mutants and genes tagged by reporter genes were developed (Goffeau, Barrell and Bussey 1996; Oliver 1996). The availability of such powerful genomic toolkits enabled researchers to investigate its ∼12 Mb (non-redundant) genome, distributed over 16 linear chromosomes (varying in size between ∼200 kb and ∼2 000 kb), inside and out. The total genome size of ∼14 Mb includes 12.07 Mb of chromosomal DNA, 85 kb of mitochondrial DNA and 6.3-kb episomal plasmids (2μ). The genome contains 6604 open reading frames (ORFs) with 79% of the ORFs verified, 11% uncharacterised and 10% regarded as dubious. A total of 1 786 ORFs are still assigned to unknown functions. The S288c genome carries 428 RNA genes (299 tRNA, 77 snoRNA, 27 rRNA, 18 ncRNA, 6 snRNA), one telomerase RNA, 295 introns in 280 genes with nine genes containing more than one intron (Engel *et al*. 2014; Peter *et al*. 2018) By comparing the S288c genome to the exponentially-growing genome sequences of other *S. cerevisiae* strains, at least 55 genes of the best-studied strains were found to be absent in S288c. There are more than 500 sets of paralogs. As more *S. cerevisiae* genomes are being sequenced, the SGD is constantly being updated and equipped with online search and analysis software. Remarkable discoveries facilitated by these genomic toolkits include the unravelling of the genetic and protein interaction networks − a prelude to full comprehension of the yeast interactome.

These highly useful resources and supporting frameworks developed for *S. cerevisiae* will increasingly open-up opportunities to accelerate research into non-model *S. cerevisiae* strains, other members of the *Saccharomyces sensu stricto* group and non-*Saccharomyces* yeasts. For example, these meta genomics, transcriptomics and proteomics tools − spearheaded by S288c− could shed a brighter light on the natural history of *S. cerevisiae*: how the life cycle of native strains progress in the wild; how they interact with other microbes in natural habitats; and what the extent of strain variation is in those natural habitats. Such information could help population biologists to conduct *reverse ecology* and to establish more definitive lineages within domesticated and wild yeast populations (Liti 2015). The efforts of the yeast research community will be well supported by applying these multi-omics and synthetic genomic technologies to non-*Saccharomyces* wine yeasts, such as *Torulaspora delbrueckii, Pichia kluyveri, Lachancea thermotolerans, Candida/Metschnikowia pulcherrima* and *Hanseniaspora uvarum*.

In summary, *S. cerevisiae* celebrity strains are the trendsetting *mannequins* modelling the future world of yeast-related discoveries, applications and innovations. Research findings with these supermodel strains could assist to extend foundational knowledge and novel technologies to non-model *S. cerevisiae* strains and non-*Saccharomyces* yeasts.

### Yeasts from nowhere

**Figure 5.**
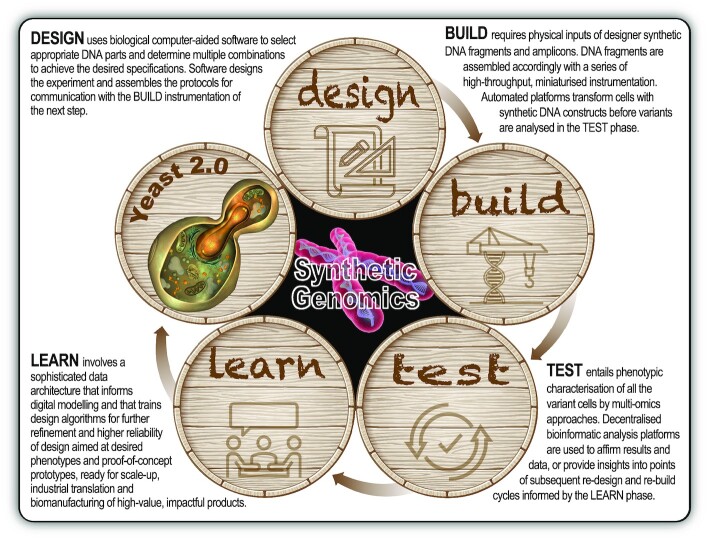
The *design-build-test-learn* (DBTL) biological engineering cycle. Application of the DBTL cycle can be accelerated in high-throughput, automated biofoundries with robotic workflows and technology platforms in Synthetic Biology. Recent rapid advances in high-throughput DNA sequencing (*reading*) and DNA synthesis (*writing* and *editing*) techniques are enabling the design and construction of new biological parts (genes), devices (gene networks) and modules (biosynthetic pathways), and the redesign of biological systems (cells and organisms) for useful purposes.

*Yeast avatars* are prototypic creations of Synthetic Biology and, in extreme instances, might even be viewed by some as *yeasts with no ancestry* − computer-designed yeasts from *nowhere*. With the confluence of modern-day biomolecular sciences, information technology and engineering, the DNA of yeasts can now be redesigned, reinvented, rewritten and edited with astounding precision (Duan *et al*.,^2010^; Gibson and Venter 2014; Lajoie *et al*. 2013; Mercy *et al*. 2017). Engineering the biology of model and non-model yeast strains (including clonal variants of natural isolates, mutants, hybrids and genetically-engineered GM strains) with laser-sharp accuracy can stretch the realms of possibility in yeast research and wine yeast innovation. By applying basic engineering principles (involving the classic rational *design*, *build*, *test* and *learn* cycle) in high-throughput, automated biofoundries with robotic workflows and technology platforms (Chao *et al*. 2017; Hillson *et al*. 2019; Walker and Pretorius 2018), the speed with which synthetic and semisynthetic prototypes can be developed, is accelerating at break-neck pace (Fig. 5). These biofoundry-based workflows encompass computational design of DNA genetic parts, physical assembly of designed DNA parts, prototyping and testing performance of designs in living cells followed by applying modelling and computational learning tools to inform the design process. Iterations of the *design-build-test-learn* cycle in biofoundries result in genetic designs that aim to fulfil the design specifications. For the time being, most of the Engineering Biology research into eukaryotes is focussed on *S. cerevisiae*, primarily to unearth more secrets of the innerworkings of this supermodel yeast as a prelude to expanding that knowledge to other *Saccharomyces* and non-*Saccharomyces* yeasts and to push the performance of industrial strains to greater heights.

Numerous game-changing technologies and milestone breakthroughs that followed the first yeast transformation in 1978 and release of the first whole genome sequence of *S. cerevisiae* in 1996, hastened the capacity to analyse and manipulate the yeast genome with remarkable success. The wealth of genomic data generated in this way, ushered yeast research into an age of Synthetic Biology where genetic variation is rationally designed, and evolution harnessed and fast-tracked. At the core of this new era of Engineering Biology lies hundreds of publicly-available yeast genome sequences and the capacity to *de novo* synthesise genes, chromosomes and genomes (Annaluru *et al*. 2014; Mitchell *et al*. 2017; Pennisi 2014; Richardson *et al*. 2017; Shen *et al*. 2017; Wu *et al*. 2017; Xie *et al*. 2017; Zhang *et al*. 2017); to edit DNA with *Clustered Regularly Interspaced Short Palindromic Repeats* (CRISPR-Cas9, CRISPR-CPF1 and other variations) technologies; and to generate large-scale genetic diversity with *Synthetic Chromosome Rearrangement and Modification by LoxP-mediated Evolution* (SCRaMbLE) via *Cre*-recombinase induction (Blount *et al*. 2018; Hochrein *et al*. 2018; Jia *et al*. 2018; Liu *et al*. 2018; Luo *et al*. 2018b; Shen et al. 2016, 2018; Wu *et al*. 2018;).

In much the same way as a genome sits at the heart of a yeast cell, the synthetic Sc2.0 genome occupies the centre of the application of CRISPR and SCRaMbLE technologies to karyotype engineering, genetic variant generation and protype strain development. The Sc2.0 genome was designed to probe some vexing questions about the fundamental properties of chromosomes, genome organisation, chromosome number, gene content and annotation; function of RNA splicing; the extent to which small RNAs play a role in yeast biology; the distinction between prokaryotes and eukaryotes; and questions relating to genome structure and evolution, while recognising that the eventual ‘synthetic yeast’ being designed and refined could ultimately play an important practical role (see www.syntheticyeast.org).

The first draft of the 16 synthetic chromosomes is now complete but there remains work to be undertaken to ensure that the growth of a strain carrying these redesigned chromosomes in a single cell would be on par with that of the original strain (Pretorius and Boeke 2018; Sliva *et al*. 2015). The Sc2.0 genome was designed to contain specific base substitutions within some of the ORFs to accommodate desirable enzyme recognition sites or deletions of undesirable enzyme recognition sites. This designer genome also includes recognisable PCRtags [short recoded sequences within certain ORFs to enable a polymerase chain reaction (PCR)-based assay] so that the synthetic DNA can be differentiated from native DNA. Other important variations between the Sc2.0 genome and that of the native strain are the addition of multiple LoxPsym sites for future genome reshuffling purposes; all TAG stop codons were recoded to TAA to free up one codon for future inclusion of unusual amino acids into *new-to-nature* proteins and enzymes; all repetitive and dispensable sequences like the five families Ty retrotransposons [a total of ∼50 copies each flanked by long terminal repeat (LTR) sequences], pre-tRNA and pre-mRNA introns, subtelomeric regions and silent *HML* and *HMR* mating-type loci were omitted from the design; and all tRNA genes were relocated to a novel neochromosome (Richardson *et al*. 2017). The decision to delete the retrotransposons and their LTRs from the design was to remove as much dispersed repetitive DNA as possible from the genome, thereby potentially delivering a more stable synthetic genome free of mobile elements. The pre-mRNA introns were accurately deleted from the design, excepting (for now) those genes with evidence of fitness defects caused by intron omission (Richardson *et al*. 2017). For example, the *HAC1* intron, which uses separate splicing mechanisms and is known to play a critical role in the unfolded protein response, was retained in the design. The rationale for the relocation of all tRNA genes to a specialised neochromosome encoding only tRNA species was based on the fact that tRNA genes lead to genome instability by replication fork collapse (Richardson *et al*. 2017). The latter might be caused by a collision with tRNA polymerase PolIII and/or the formation of R-loops at actively-transcribed tRNA genes, which impede the replication fork in a polar manner, ultimately causing replication fork stalling and subsequent repair through recombination.

The draft set of 16 synthetic Sc2.0 chromosomes has already been put to good use to answer some profound fundamental biological questions, thereby helping researchers to understand what basic genomic features and genetic combinations are essential for cell viability. For example, one of the most fundamental characteristics of the *S. cerevisiae*’s genome queried was the number of chromosomes in each cell and the and industrial relevance thereof (Gorter de Vries, Pronk and Daran 2017). Chromosome number varies wildly across eukaryotes. Thus, two basic questions to ask would be why does a haploid *S. cerevisiae* strain distribute its genomic DNA along 16 chromosomes, and how well would it tolerate a change in its chromosome number without substantial changes to its genome content. Two independent studies were conducted to answer these intriguing questions. In one study, CRISPR-Cas9-mediated genome editing was used to fuse *S. cerevisiae*‘s chromosomes and generate a near-isogenic series of strains with progressively fewer chromosomes until the whole genome was compacted into two chromosomes (Luo *et al*. 2018a). These researchers found that as the number of chromosomes dropped below 16, spore viability decreased dramatically. However, homotypic crosses between pairs of strains with 8, 4 and 2 chromosomes produced good spore viability, demonstrating that eight chromosome fusion events suffice to isolate strains reproductively. In another study, the DNA of *S. cerevisiae*’s 16 native linear chromosomes were squeezed into a single chromosome by successive end-to-end chromosome fusions and centromere deletions (Shao *et al*. 2018). Although the strain carrying the giant chromosome supported viability, it did show reduced growth and less competitiveness across different culturing conditions. Nevertheless, these karyotype engineering experiments uncovered the surprising insight that *S. cerevisiae* copes remarkedly well with one or two mega chromosomes instead of sixteen. These two studies indicated that chromosome number seems to reflect ‘accidents of genome history’, such as telomer–telomer fusions and genome duplication events (Luo *et al*. 2018a; Shao *et al*. 2018).

The learnings from the Sc2.0 project are also advancing the frontiers of researchers’ understanding of how they can expand the genetic range of yeast prototypes and reshuffle the genome for industrial applications. To improve the complex rearrangements achieved by SCRaMbLE, the original method has been adapted to accelerate and expand the diversity of prototypical strains and to rapidly identify them. Two such adaptations are MuSIC (*Multiplex SCRaMbLE Iterative Cycling*) and ReSCuES (*Reporter of SCRaMbLEd Cells using Efficient Selection*) (Jia *et al*. 2018; Luo *et al*. 2018b). In one example, MuSIC was used to put yeast strains through five cycles of SCRaMbLE under selective pressure, thereby driving increases in catenoid titres. In another example, ReSCuES was utilised to quickly screen for successfully SCRaMbLEd cells by integrating two selective markers, one of which was functional before SCRaMbLE and one only after the *Cre* recombinase inverted their orientation (Luo *et al*. 2018b). To enhance recombination control, red light was employed to control *Cre* (Hochrein *et al*. 2018). In addition, the principles of SCRaMbLE have been extended from an *in vivo* to an *in vitro* technique. It also appears that mating *S. cerevisiae* carrying synthetic Sc2.0 chromosomes with wild-type *S. cerevisiae* strains or closely related *Saccharomyces* species can overcome the potential for highly-desirable recombination events even if they impose severe growth defects.

These improvisations boosted the SCRaMbLE toolkit to rapidly generate an enormous amount of genetic variation for both basic research and applied industrial purposes. For example, by applying SCRaMbLE in a test tube rather than a cell, a pathway or a set of genes can now be removed from the complexity of the genome and used to quickly generate huge prototype variations in a single pathway (Liu *et al*. 2018; Wu *et al*. 2018). In other instances, when SCRaMbLE was applied to strains containing the synthetic version of Chromosome V, strains were generated with marked increases in their capacity to produce violacein and penicillin, or in their capacity to utilise xylose as a carbon source (Blount *et al*. 2018).

SCRaMbLE is now also being applied to hundreds of different *S. cerevisiae* strains with distinctive phenotypes that provide an advantage in a specific environmental niche or industry, such as baking, brewing and winemaking. These phenotypic differences are the direct result of specific genetic variation among strains and this can range from single nucleotide polymorphisms (SNPs), to the presence of strain-specific genes or gene clusters (Borneman *et al*. 2011; Borneman, Pretorius and Chambers 2013a; Borneman, Schmidt and Pretorius 2013b). The presence or absence of these genes among strains can have remarkable phenotypic consequences, including providing strains with the ability to synthesise vitamins or to endure specific types of stress or inhibitory compounds. To provide greater insight into the role of these strain-specific genes, researchers identified over 200 kb of non-repetitive DNA, encoding 75 ORFs, which exist across the breadth of strain-specific ORF diversity of the *S. cerevisiae* pan-genome, but which are absent from the laboratory strain used for Sc2.0 (Borneman *et al*. 2011; Borneman, Pretorius and Chambers 2013a; Borneman, Schmidt and Pretorius 2013b). These sequences have been synthesised and assembled into a neochromosome. This pan-genome neochromosome is now being analysed to determine the phenotypes that it can impart in a laboratory strain background, while also providing a resource for introducing additional variation into the Sc2.0 genome through processes such as SCRaMbLE.

These developments bode well for future applications of SCRaMbLE for the improvement of a diverse range of unrelated metabolic pathways that are of commercial interest, including pathways that could enhance the aroma of wine or lead to a reduction in the level of alcohol concentration in light-bodied wines (Goold *et al*. 2017; Kutyna and Borneman 2018; van Wyk, Kroukamp and Pretorius 2018). The *in vivo* and *in vitro* prototyping of rearranged pathways involving pan-genomic neochromosomes or *avatar* strains of *S. cerevisiae* with altered karyotypes will undoubtedly accelerate the uncovering and harnessing of the full potential of diversity found in the yeast genome.

Another frontier is currently igniting the imagination of some adventurous synthetic biologists, is the potential to generate synthetic yeast organelles. Compartmentalisation is a fundamental mechanism for eukaryotic cells to segregate and sequester valuable biomolecules (Pretorius 2000). Yeast organelles compartmentalise important cellular processes, such as energy production in mitochondria and the decoration of proteins with sugars and their packaging into membrane-bound vesicles for intracellular sorting and secretion by the Golgi apparatus. The ability to concentrate substrates together for particular reaction pathways, and separate them from competing reactions, enables highly-efficient biosynthesis of valuable compounds (e.g. desirable flavour-active compounds). The separation of toxic chemicals or production of chemical gradients allows unique reactions to occur that would be impossible in a single compartment. However, despite the benefits of compartmentalisation, natural organelles are limited by numerous demands imposed by yeast cells or the convoluted evolutionary trajectories that created them. In the medium to long future, the untapped potential of compartmentalisation in yeast cells is set to be unleashed by the re-engineering of existing organelles and building completely new organelles from scratch.

These transformative synthetic genomic tools and concomitant prototypical yeast *avatars* will only translate into practical applications in the winery if they can tread lightly over the GMO quicksand where consumer perceptions are their reality regardless of numerous scientific reassurances of safety. In this quagmire that has trapped so many GM food products, it has been shown repeatedly that it is irrelevant whether a product is safe if people refuse to consume it for psychosocial or cultural reasons. Although CRISPR editing technologies provide researchers with the ability to redesign − with surgical precision − specific genes and gene networks without having to introduce foreign DNA from other organisms, it remains to be seen whether consumers will take advantage of what *avatar* wine yeasts are able to offer them. At present, producing commercial wine with *avatar* strains seems distant. So, what will it take to persuade the public that the benefits of, for example, an *avatar* yeast strain capable of producing low-alcohol wines with superior flavour profiles, outweighs any safety risks and fears for the erosion of the sacrosanct concept of *terroir*? Understanding what drives public sensitivities and consumer perceptions, preferences and purchase decisions is key to answering this question in the context of an archetypal traditional product such as wine with such strong territorial and socio-cultural connotations.

## LINKING YEAST WITH WINE QUALITY AND CONSUMER GRATIFICATION

We humans like the good things in life. Our dogged search for pleasure inspires and motivates us and drives industries such as the wine sector to meet demand. But that demand is changing. At any time on any given day, someone somewhere is enjoying a good glass of wine. But what do we mean by good? Is it the appearance, aroma, taste or texture? Professional wine tasters would say all of the above. The reality is more complex. Last century, it was acceptable for wines to just be *good*. Today, wines have to be *great* in order to stand out in overcrowded consumer markets. *Good* is not enough when your competition is 30 billion litres of wine and an annual global surplus of ∼15%. *Great* requires a total commitment to innovate. Today's fickle consumer expects their wine to provide a sensory experience; to be safe; to be produced in an environmentally sustainable way; and to measure up to an indefinable mystique. Inventive winemakers recognise that everything worthwhile in today's consumer market is uphill and, to successfully meet the ever-changing demands of wine drinkers, ingenuity and innovation are required − every step of the way, every day, all the way. Innovative winemakers realise that the success of their carefully crafted wines is often at the mercy of increasingly tech-savvy consumers who communicate their likes and dislikes globally and instantaneously via websites, blogs and social media (Jagtap *et al*. 2017). In this *click-like/dislike-share* era, consumers expect more than products and services in return for their money. They expect an experience that embraces a *value chain* from where a grape is grown to how the wine is produced and packaged. For winemakers, innovation means understanding their consumers and exceeding their expectations. They must anticipate what wine drinkers of today and tomorrow want to see, smell, touch, taste, feel and hear. To succeed in doing so, winemakers increasingly rely on rigorous science and technical know-how to create great experiences with wines. Truly great wines are born of great marriages between grape variety and *terroir* on the one hand, and technology, innovation and craftsmanship on the other. This century, winemaking has become an industry where art meets science − including the science that underpins yeast biology and the fermentation process. Backed by robust research and evidence-based data, winemakers can put their own individual stamp on the production chain by making well-informed and insightful decisions as to what yeast strain or combination of yeast strains will best guide their products to market success (Dzialo *et al*. 2017; Gallone *et al*. 2016; Hyma *et al*. 2011; Pretorius and Bauer 2002; Swiegers *et al*. 2005).

The expectations and preferences of wine drinkers often use the terms *quality* and *value* when they compare different wines for purchase. In this context, the term *quality* relates to the ‘intrinsic’ quality of the wine, meaning how the wine gratifies on appearance, the nose and the palate, as well as the perceived value. When consumers use the term *value*, they usually refer to both the *intrinsic value* and the *image* of a wine in relation to the price (Pretorius and Høj 2005). The image of a product depends on how a wine is marketed, the origin, regionality and *terroir* of a wine, how environmentally-sound the winery's practices are, how many medals have been awarded at wine shows, how high sommeliers and other market influencers rate a wine, and price. Consumers will consider a wine high in value if the product is sensorially pleasing and recognisable, and perceived as high in image at a competitive price.

**Figure 6.**
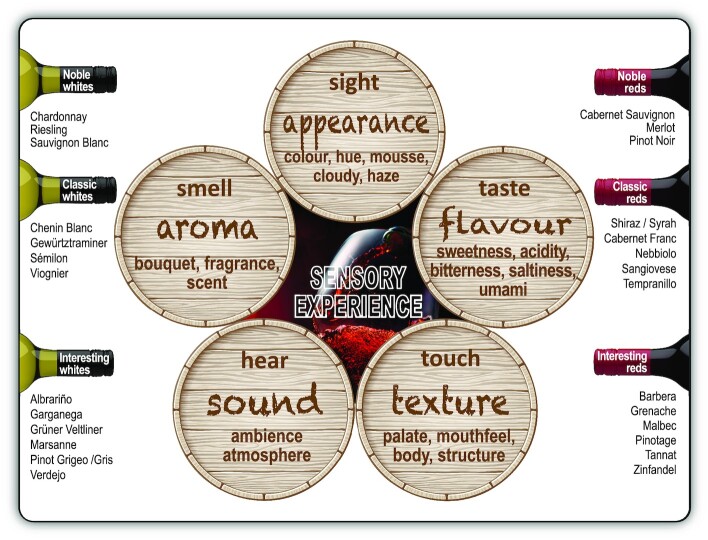
The sensorial quality of wine involves all five senses sight, smell, taste, touch and sound. The *appearance* of wine can be affected by cloudiness, haziness, a deposit in the bottom of the glass, and the depth of colour, hue, mousse). The *smell* of wine refers to both the *aroma* and the *bouquet*. A wine's *palate*, *taste* and *touch* refer to its *flavour* and *mouth-feel*.

In terms of how much *value* consumers attach to a product, the single most important factor that defines a consumer's perception of wine is its organoleptic quality, which involves four senses, i.e. sight, smell, taste and touch (Fig. 6). Professional wine tasters would normally describe what they sense by referring to a wine's appearance, aroma, flavour and texture (Pretorius 2016). *Appearance* (sight ‒ e.g. cloudy, hazy, deposit in the bottom of the glass, depth of colour, hue, mousse), *nose* (smell ‒ e.g. aroma and bouquet) and *palate* (taste and touch ‒ flavour and mouth-feel). The term *aroma* is typically used to describe the fragrant smell of a young, fresh wine whose *primary* aromas and upfront fresh-fruit notes originate during yeast fermentation. *Bouquet* is the term for more mature wines that are less fresh but more complex thanks to *secondary* aromas (e.g. stewed fruit notes) stemming from oak maturation and *tertiary* aromas (e.g. dried fruit and honey notes) forming during bottle ageing. The term *flavour* refers to sweetness, acidity, bitterness, saltiness and the taste of umami. The descriptor of *mouth-feel* relates to the texture and body of wine as affected by factors such as alcohol strength (sensation of warmth) and tannins (drying sensation). The *structure* of a wine describes its acidity, sweetness, bitterness (occasionally), tannin (in red wine), alcohol, palate weight and length, mouth-feel, mousse (in sparkling wine), as well as the intensity of fruit aroma and flavour, and complexity (diversity and layers of flavour).

A wine's sensory quality is largely determined by the presence of desirable flavour compounds and metabolites in a well-balanced ratio, and the absence of undesirable ones. The *absolute* and *relative* concentrations of these flavour-active compounds are important to shape a wine's smell and taste. The choice of a *S. cerevisiae* starter strain in single-strain fermentations, or combination of *Saccharomyces* and/or non-*Saccharomyces* yeasts in multi-strain and multi-species fermentations, is by far the most cost-effective, time-efficient and flexible way to shape the aroma and flavour according to changing consumer preferences than to replant a vineyard, manage climatic conditions or adapt viticultural practices (Jolly *et al*. 2014). This is the primary driver behind the never-ending search for naturally-occurring yeast variants inhabiting different *terroirs*, the genetic improvement of well-characterised yeast strains and the exploration of various combinations of compatible *Saccharomyces* and non-*Saccharomyces* starter yeast cultures aimed at meeting the shifting consumer preferences in specific segments of the market.

### Flavour-active non-*Saccharomyces* wine yeasts

To meet the preferences of consumer markets where the appeal of *terroir, typicity* and wine *complexity* dominates purchasing decisions, a growing number of winemakers are experimenting with non-*Saccharomyces* yeast starter cultures. Some winemakers pursue this goal by conducting spontaneous fermentation while others, who wish to avoid the risks of sluggish/stuck ferments and spoilage often associated with uninoculated fermentations, search for co-culturing candidate yeasts in the biodiversity treasure trove of their vineyards and culture collections. There is a growing list of these so-called *unconventional* yeasts as potential candidates for co-culturing with stalwart *S. cerevisiae* wine strains. So far, the non-*Saccharomyces* species that received most attention in this regard include *Candida stellata*, *Debaryomyces vanriji*, *Hanseniaspora uvarum*, *Hanseniaspora vineae*, *Kazachstania gamospora*, *Lachancea thermotolerans*, *Metschnikowia pulcherrima*, *Pichia fermentans*, *Pichia kluyveri*, *Rhodotorula mucilaginosa, Schizosaccharomyces pombe*, *Torulaspora delbrueckii*, *Williopsis saturnus* and *Zygotorulaspora florentina* (Jolly *et al*. 2014) These multi-species fermentation strategies seem to be an effective way to mimic flavour-diversity and product complexity outcomes obtained by successful spontaneous fermentation without forfeiting fermentation performance, reliability and overall wine quality.

In addition to the of use these non-*Saccharomyces* yeasts in co-cultured ferments, winemakers also apply a range of different inoculation regimes (Pretorius 2000, 2016, 2017a). Some inoculate their ferments sequentially − first with the non-*Saccharomyces* species and allow those yeasts to participate in the fermentation process before they inoculate the fermenting must with a selected *S. cerevisiae* starter culture strain(s). In this way, the non-*Saccharomyces* species have sufficient time to contribute to the sensory profile of the wine before they are constrained by high alcohol concentrations stemming from fast-fermenting alcohol-tolerant *S. cerevisiae*. The potential down-side of such a sequential inoculation regime is that the non-*Saccharomyces* could use up some nutrients that are essential to *S. cerevisiae* during the later stages of the fermentation process. It is for that reason that some winemakers choose to co-inoculate non-*Saccharomyces* and *S. cerevisiae* starter cultures at the same time. However, there are also variations within this strategy. Winemakers could opt for simultaneous inoculation of non-*Saccharomyces* and *S. cerevisiae* starter cultures but start off by using a much lower dosage (cell count) of *S. cerevisiae*, thereby giving some ‘first-mover’ advantage to the slower non-*Saccharomyces* fermenters in the early phase of fermentation. The initial ratio of non-*Saccharomyces* versus *S. cerevisiae* yeasts in the starter culture is therefore another important decision point to conduct controlled co-cultured wine fermentations. These choices will depend on the specific species and strains of non-*Saccharomyces* and *S. cerevisiae* (and their state of metabolic activity) in the co-cultured ‘yeast starter cocktails’ (Pretorius 2016). It is also important that the selected non-*Saccharomyces* species be compatible (non-antagonistic) with the companion *S. cerevisiae* strain, whether sequentially or simultaneously inoculated. Another point to consider is whether the non-*Saccharomyces* species can be produced as active dried cultures. It is well-known that yeast manufacturers often struggle to optimise the growth conditions and/or drying conditions in their factories for some of these non-*Saccharomyces* yeasts. Despite these challenges, there are now commercialised yeast starter culture products available to winemakers and some of these multi-species and multi-strain ‘yeast blends’ are pre-packaged in optimal ratios for fermentation performance and sensory outcomes.

Varying degrees of success with multi-species ferments have been reported. Generally, a decrease in volatile acidity (responsible for vinegary aromas) is observed with such co-culturing because several non-*Saccharomyces* species (e.g. *T. delbrueckii*) produce low concentrations of acetic acid (Bely *et al*. 2008). Multi-species ferments also tend to produce an increase in ester production, especially with *Hanseniaspora* and *Metschnikowia* species (Martin *et al*. 2018). Some non-*Saccharomyces* species secrete flavour-enhancing enzymes, such as *β*-glucosidase and *β*-lyase (Belda *et al*.2016a). It is important to note that there are significant strain variations within the same non-*Saccharomyces* species in terms of their capacity to influence the sensory profile of the final product.

Several studies reported positive sensory outcomes when certain non-*Saccharomyces* species were paired up with *S. cerevisiae* in multi-species ferments. For example, *D. vanriji* (Garcia *et al*. 2002) was reported to increase the concentration of geraniol in Muscat wines while *K. gamospora* (Dashko *et al*. 2015) was found to increase the levels of phenylethyl alcohol and phenylethyl acetate in Ribolla Gialla. In another study, *H. vineae* also produced increased levels of phenylethyl acetate in Blobal (Viana *et al*. 2011). *P. kluyveri* is known to increase the concentration of volatile thiols in Sauvignon Blanc (Anfang, Brajkovich and Goddard 2009). Increased ester concentrations were observed in Cabernet Sauvignon, Riesling, Merlot and Sangiovese when *L. thermotolerans* (Gobbi *et al*. 2013), *M. pulcherrima* (Röcker *et al*. 2016), *T. delbrueckii* (Renault *et al*. 2015) and *Z. florentina* (Lencioni *et al*. 2016), respectively, were used in co-cultured fermentations. *W. saturnus* was reported to increase isoamyl acetate in Emir wines (Tanguler 2013). *S. pombe* is used to remove the sharp-tasting malic acid and decrease the ‘tartness’ in Ezerfürtű wine (Yokotsuka *et al*. 1993). *R*. *mucilaginosa* was reported to improve the aroma of Ecolly wine (Wang *et al*. 2017). These are only a few examples of the benefits of including non-*Saccharomyces* species in conjunction with *S. cerevisiae* wine strains in multi-species ferments. There is no doubt that the hunting for beneficial non-*Saccharomyces* companions for *S. cerevisiae* wine yeasts will continue and that biodiversity prospecting in vineyards will unearth more interesting yeasts in the years to come.

### Flavour-active *Saccharomyces* wine yeasts

Since cracking the genetic code of the first wine yeast strain (AWRI1631) in 2008, the genomes of several other widely used commercial wine yeast strains − including AWRI1796, EC1118, QA23, VIN7, VIN13 and VL3 − were sequenced and compared with the genomes of laboratory strains of *S. cerevisiae* (S288c and Sigma1278b) as well as genomes of commercial *Saccharomyces* strains used in the baking, brewing, biofuel, ragi and saké industries (Borneman *et al*. 2008; Galeote *et al*. 2010). An additional stretch of ∼200 kb of DNA present in some of these industrial strains, but found to be lacking in the laboratory strains include strain-specific loci. These loci reside in the hypervariable subtelomeric regions and, in some cases, they distinguish specific classes of industrial strains. For example, a member of a subtelomeric three-gene cluster, the *RTM1* locus, is chiefly present in ale and distilling strains and to some extent, in strains that carry a set of genes specific to wine yeasts (Borneman *et al*. 2008). This wine-specific suite of genes comprises a second industry-defining locus, which consists of a cluster of five genes, displaying strain differences in copy number, genomic location and gene order most likely due to mobilisation into, and throughout, wine-strain genomes as a circular intermediate via an unknown process (Borneman *et al*. 2008). The third industry-specific locus entails the evolutionary differences in biotin prototrophy amongst certain industrial strains. More specifically, while the majority wine and beer strains are biotin auxotrophs, saké strains acquired the capacity to synthesise biotin *de novo* over time, presumably because of evolutionary pressures in the low-biotin fermentations of saké mash (Borneman *et al*. 2008).

Three wine-strain-specific genes were also identified: (i) the *FSY1* gene encoding a H^+^/fructose symporter; (ii) two paralogues, *MPR1* and *MPR2*, conferring resistance to l-azetidine-2-carboxylic acid; (iii) and the *β*-lyase encoding gene, *IRC7* (Borneman *et al*. 2008). It is reasonable to argue that an effective H^+^/fructose symporter could provide a selective advantage to strains expressing *FSY1* in highly concentrated mixtures of glucose and fructose during the fermentation of grape must. Wine strains that are capable of consuming fructose efficiently are less likely to produce sluggish or stuck fermentations during vintages in which heat waves distort the usual equilibrium between glucose and fructose in grape juice. Strains that carry the *MPR*-family paralogues cope better with stressful fermentation conditions because they are able to decrease the toxic effects of reactive oxygen. It is believed that the fermentation performance of *MPR*-carrying strains is more robust. Whereas, whilst the *FSY1* and *MPR1/2* genes are thought to convey fermentation robustness and performance, the *IRC7* gene might be associated with aroma enhancement in wine. *IRC7*-expressing strains seem to release more volatile thiols during fermentation, thereby increasing the fruitiness of wine. Paradoxically, the functional version of *IRC7* is rarely found in wine strains (Belda *et al*.2016b).

Insightful observations were also made by analysing the genomes of some members of the *Saccharomyces sensu stricto* clade. This clade consists of the *S. cerevisiae* complex (comprising well-defined ‘pure’ lineages based strictly around geographic or industrial parameters, and mosaic strains that appear to be the result of outcrossing between multiple pure lineages), and seven distinct *Saccharomyces* species (*S. arboricolus*, *S. cariocanus*, *S. eubayanus*, *S. kudriavzevii*, *S. mikatae*, *S. paradoxus*, and *S. uvarum*). For instance, comparative genomic analyses revealed that the thiol-releasing wine yeast, VIN7, has an allotriploid hybrid genome with *S. cerevisiae* and *S. kudriavzevii* origins (Borneman *et al*. 2012). That explained the genetic basis of this VIN7’s unique capacity to produce wines with a distinctive guava-like aroma. However, additional analyses of more natural hybrids like VIN7 are required to uncover more genes responsible for distinct flavours before bioengineers would be able to construct complex aroma-enhancing metabolic pathways in yeast strains customised for specific wine styles.

In this regard, with the construction of the first semisynthetic wine strain it was demonstrated that customised flavour-activity falls with the realm of possibility (Lee *et al*. 2016; Kutyna and Borneman 2018). A haploid wine strain (AWRI1631) of *S. cerevisiae* was equipped with a biosynthetic pathway, which consists of four separate enzymatic activities required for the production of the raspberry ketone, 4-[4-hydroxyphenyl]butan-2-one. This phenylpropanoid is the principal aroma compound found in raspberries and it is also present, to a lesser extent, in blackberries, grapes and rhubarb. The phenylpropanoid pathway begins with the conversion of phenylalanine to *p*-coumaric acid via cinnamate or directly from tyrosine to *p*-coumaric acid. Conversion of *p*-coumaric acid to raspberry ketone requires three additional enzymatic steps including a condensation reaction between coumaroyl-CoA and malonyl-CoA. To construct a raspberry ketone biosynthetic pathway in a haploid wine strain (AWRI1631), the following codon-optimised genes were synthesised and integrated into its *HO* locus: the phenylalanine ammonia lyase from an oleaginous yeast, *Rhodosporidium toruloides*; the cinnamate-4-hydroxylase from *A. thaliana*; and the coumarate CoA ligase 2 gene from parsley, *Petroselinum crispum*, fused by a rigid linker to the benzalacetone synthase from rhubarb, *Rheum palmatum*. This semisynthetic wine yeast was able to synthesise raspberry ketone at concentrations nearly two orders of magnitude above its predicted sensory threshold in Chardonnay grape juice under standard wine fermentation conditions, while retaining the ability to ferment the must to dryness (Lee *et al*. 2016).

For now, though, the practical implications of the Sc2.0 lab yeast and this raspberry flavour-active wine yeast all lie in the future. These breakthroughs can best be thought of as a *Sputnik moment* in yeast research − by itself, Sputnik did nothing except for orbiting the Earth while beeping, but it proved a concept and grabbed the attention of the world's futurists. These yeast *avatars* send a strong signal to synbio sceptics that the promise of synthetic biology can be realised in theory as well as in practice. It is a red-hot research field but it also a field prone to *hype-horror-hope* oscillations − a great deal of research remains to be done before yeast *avatars* will be used for real-world tasks in commercial wineries.

## INVENTING A BRIGHTER FUTURE FOR WINE WITH YEAST

In an era of transformative technologies, unprecedented breakthroughs and award-winning research in the converging fields of Synthetic Biology, Artificial Intelligence and Quantum Computing, we could do worse than remember that science prizes tend to be bestowed upon those researchers who have made esoteric, if profound, scientific advances in the laboratory rather than practical ones in, for example, the winery. We need to separate the justified excitement in the laboratory from the opportunistic hyperbole in the winery and marketplace.

Beyond the scholarly contributions to an academic research field there are often ‘soft’ and ‘hard’ impacts along the non-linear *inputs-activities-outputs-outcomes-impacts* innovation pathway. These two kinds of impacts can have intended and unintended consequences. In this context, *hard impacts* refer to those tangible benefits that can directly be attributed to a research initiative (e.g. the *Yeast 2.0* project) or invention (e.g. the raspberry flavour-active wine yeast) whereas *soft impacts* tend to occur via uptake and use of new ideas, knowledge or innovations by independent parties under indirect (or no) influence from the original researchers. The *agri-industrial* era is evolving into an unpredictable *bio-informational* future. It is vital that all wine industry stakeholders – researchers, industry practitioners, policymakers, regulators, commentators and consumers – stay attuned to developments in these future-shaping technologies. This includes the roll-out of algorithm-enabled automation in biofoundries focussed on accelerating and prototyping biological designs for engineering-biology applications. Stakeholders need to embrace the creative spark of uncertainty, as we all live in a world where we have to expect the unexpected. In doing so, we can ignite the ingenuity of researchers and practitioners to think anew about, for example, *microbiomes* of grapes from different viticultural sites, *signature microbial terroirs* and the co-culturing of region-specific *Saccharomyces* and non-*Saccharomyces* strains in multi-starter ferments.

As described in previous sections, vineyard- and winery-related yeasts live as members of complex multi-species microbial communities that provide robustness to environmental perturbations (*terroirs*) and extended metabolic capacities. Such naturally-occurring microbial assemblages are shaped by environmental gradients and resource availability, and are spatially and functionally organised in a way that optimises organism fitness and overall community productivity. The inherent division of ‘labour’ among microbial populations with specialised sub-functions allows communities to reduce the metabolic burden imposed on each population and to carry out tasks that no single organism could undertake. Cooperative interactions among community members in the vineyard overcome the physiological and metabolic constraints of individual microbes and allow mixed microbial populations to execute otherwise incompatible functions simultaneously. Blending the synergistic modular design of naturally-occurring microbial communities in grape must with the engineering capacity of Synthetic Biology opens up new vistas for studying, modelling and optimising spontaneous wine fermentation scenarios.

In the same way that computers are built using different specialised hardware, rationally connected to increase overall system performance, *synthetic microbial communities* can be ‘biologically wired’ to collectively perform complex higher-order tasks unattainable by using individual populations from the vineyard or grape must. By employing such a ‘plug-and-play’ approach, specialised permutations of compatible *Saccharomyces* and non-*Saccharomyces* yeast populations can be differentially combined in multi-starter ferments depending on the desired outcome from a wine quality perspective. This will allow researchers to harness synergistic wine yeast consortia properties and tap into the unparalleled functional versatility of *terroir*-specific *Saccharomyces* and non-*Saccharomyces* yeasts. Building such *synthetic yeast starter-culture consortia* for modelling purposes will require an acceleration of the current *biodiversity prospecting* in vineyards with recognisable *microbial terroir* effects and the purposeful hunting for non-*Saccharomyces* yeasts in those vineyards and associated wineries.

It won't be a surprise if concepts such as synthetic microbial consortia that include yeasts with recoded single-chromosome genomes, carrying synthetic organelles, expressing re-engineered biosynthetic pathways for highly-reactive and orthogonal cofactors, and producing ‘new-to-nature’ compounds, sound like geeky ‘sci-fi’ abracadabra to most winemakers. However, for many researchers involved in the engineering of biology, this is *science fiction* in the process of transitioning to *science fact*.

Few doubt that a wine yeast *avatar* − completely powered by a computer-designed and synthetically made genome − is possible in principle; however, the consensus is that the use of such *avatars* in commercial wine production is not imminent. Anyone counting on widespread application of wine yeast *avatars* to tantalise consumers’ taste buds and open their wallets for repeat purchases in the near future will do well by remembering this cautionary note. At the same time, it is prudent to not lose sight of the wine industry's *tradition of innovation* − a metaphorical *terroir* free from cognitive prejudices where the newest ideas in wine yeast innovation can be powered by the oldest traditions in winemaking.
